# Internalization of apoptotic cells during efferocytosis requires Mertk-mediated calcium influx

**DOI:** 10.1038/s41419-023-05925-7

**Published:** 2023-06-30

**Authors:** Susumin Yang, Chanhyuk Min, Hyunji Moon, Byeongjin Moon, Juyeon Lee, Jaeseon Jeon, Hagyeong Kwon, Deokyun Jang, Daeho Park

**Affiliations:** 1 School of Life Sciences, Gwangju Institute of Science and Technology, Gwangju, 61005 Korea; 2 Cell Mechanobiology Laboratory, Gwangju Institute of Science and Technology, Gwangju, 61005 Korea; 3 School of Electrical Engineering and Computer Science, Gwangju Institute of Science and Technology, Gwangju, 61005 Korea

**Keywords:** Apoptosis, Cell signalling

## Abstract

Phagocytosis of apoptotic cells, called efferocytosis, requires calcium inside and outside of phagocytes. Due to its necessity, calcium flux is sophisticatedly modulated, and the level of intracellular calcium in phagocytes is ultimately elevated during efferocytosis. However, the role of elevated intracellular calcium in efferocytosis remains elusive. Here, we report that Mertk-mediated intracellular calcium elevation is necessary for internalization of apoptotic cells during efferocytosis. Drastic depletion of intracellular calcium abrogated the internalization step of efferocytosis by delaying phagocytic cup extension and closure. Especially, the defect of phagocytic cup closure for internalization of apoptotic cells was caused by impaired F-actin disassembly and the attenuated interaction of Calmodulin with myosin light chain kinase (MLCK), leading to diminished myosin light chain (MLC) phosphorylation. Genetic and pharmacological impairment of the Calmodulin-MLCK-MLC axis or Mertk-mediated calcium influx also resulted in inefficient efferocytosis due to a defect in internalization of the targets. Taken together, our observations imply that intracellular calcium elevation through Mertk-mediated calcium influx facilitates efferocytosis by inducing myosin II-mediated contraction and F-actin disassembly required for internalization of apoptotic cells.

## Introduction

Efferocytosis is a type of phagocytosis that removes endogenous particles, namely, apoptotic cells [1]. Phagocytes distinguish apoptotic cells from other cells not to be phagocytosed through interactions between ligands on apoptotic cells and their receptors on phagocytes [2–4]. Studies conducted over the past several decades have identified various molecules involved in recognition of apoptotic cells by phagocytes and delineated their signaling pathways. One of them is phosphatidylserine (PS), which is exposed on apoptotic cells and functions as a pivotal ligand during efferocytosis [2, 5]. It is directly or indirectly recognized by redundant engulfment receptors on phagocytes [6–11], one of which is Mertk, which indirectly recognizes PS on apoptotic cells. Mertk is a member of the TAM receptor family and recognizes PS on apoptotic cells via bridging molecules such as Gas6, which binds to both PS and Mertk [12]. Mertk also biochemically interacts and thereby functionally co-operates with Tim-4, a PS receptor (PSR) that directly binds to PS, during efferocytosis. In Mertk and Tim-4-mediated efferocytosis, apoptotic cells secured on phagocytes through Tim-4 are readily recognized by Mertk, which subsequently transduces signals into phagocytes to internalize apoptotic cells [13–15].

In internalization of apoptotic cells, Rac1 is activated by signaling downstream of engulfment receptors and plays a central role by inducing actin cytoskeletal rearrangement during efferocytosis [16–18]. In particular, spatiotemporal regulation of Rac1 activity is essential for phagocytic cup extension (pseudopod extension) and closure (pseudopod sealing) causing internalization of apoptotic cells during efferocytosis [19]. Activation of Rac1 causes actin polymerization to form and extend the phagocytic cup, but Rac1 should be inactivated for phagocytic cup closure. Inactivation of Rac1 immediately following its activation leads to F-actin disassembly and myosin light chain (MLC) phosphorylation, inducing myosin II-mediated contraction required for phagocytic cup closure, which eventually results in internalization of apoptotic cells [20–22].

Efferocytosis, like other cellular processes, requires calcium to efficiently phagocytose apoptotic cells [23]. Both extra- and intracellular calcium are essential for efferocytosis, and disturbance of proteins involved in calcium influx impedes efferocytosis [24–28]. Extracellular calcium is necessary for binding of PS receptors and bridging molecules to PS [29]. It also serves as a source of intracellular calcium. Extracellular calcium enters phagocytes through store-operated calcium entry (SOCE), causing intracellular calcium elevation during efferocytosis [27]. Mertk mediates SOCE through the PLCγ1-IP_3_R axis during efferocytosis. Upon binding of PS on apoptotic cells to Mertk, PLCγ1 and IP_3_R are sequentially activated, leading to release of calcium from the endoplasmic reticulum and induction of the Orai1-Stim1 interaction, which provokes SOCE. These processes eventually increase the intracellular calcium level in phagocytes during efferocytosis. During efferocytosis, the intracellular calcium level in phagocytes is also elevated through Drp-1-mediated mitochondrial fission blocking Mcu-1-mediated mitochondrial calcium sequestration, which facilitates continuous removal of apoptotic cells [28]. Nevertheless, the roles of intracellular calcium in efferocytosis are not completely explored.

In this study, we found that intracellular calcium affects efferocytosis by modulating the internalization step of efferocytosis. Intracellular calcium depletion in phagocytes impaired efferocytosis without affecting the binding activity, Rac1 activation inducing F-actin formation, and phagolysosomal acidification of phagocytes. However, intracellular calcium depletion inhibited MLC phosphorylation and F-actin disassembly at the phagocytic cup, which delayed internalization of the targets. Genetic and pharmacological impairment of an MLC phosphorylation pathway or Mertk-mediated calcium influx also increased the time required for internalization of apoptotic cells during efferocytosis. Collectively, our findings suggest that elevation of the intracellular calcium level through Mertk-mediated SOCE promotes F-actin disassembly and myosin II-mediated contraction required for internalization of apoptotic cells during efferocytosis.

## Results

### Intracellular calcium affects efferocytosis by modulating internalization of the target

**Fig. 1:**
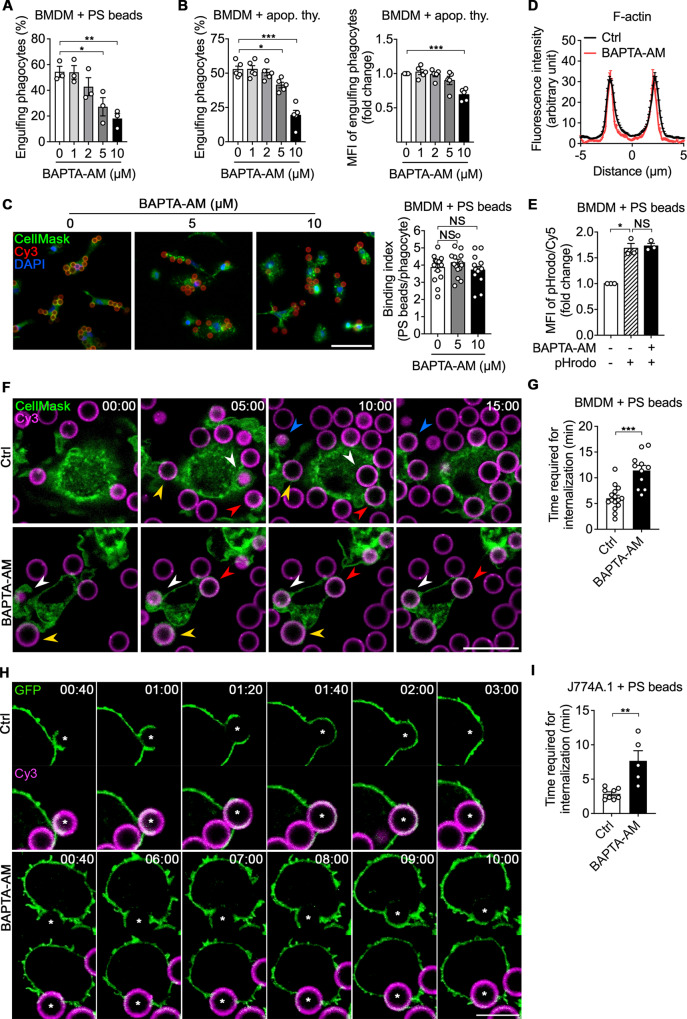
Intracellular calcium depletion delays internalization of targets during efferocytosis. **A**, **B** BMDMs pretreated with the indicated concentrations of BAPTA-AM for 30 min were incubated with Cy3-labeled PS beads (**A**) or TAMRA-stained apoptotic thymocytes (**B**) for 15 min, washed to remove unbound targets, trypsinized, and analyzed by flow cytometry. Data are mean ± s.e.m. from n = 3 (**A**) and n = 5 (**B**) independent experiments. *P < 0.05; **P < 0.01; ***P < 0.001; one-way ANOVA. **C** CellMask-stained BMDMs pretreated with the indicated concentrations of BAPTA-AM for 30 min were incubated with Cy3-labeled PS beads at 4 °C for 2 h, extensively washed with PBS to remove unbound targets, fixed, and stained with DAPI. Bound targets were observed by fluorescence microscopy (left) and quantified (right). Scale bar, 50 µm. Data are mean ± s.e.m. from *n* = 15 randomly acquired images. NS, not significant; one-way ANOVA. **D** BMDMs treated with or without BAPTA-AM were incubated with Cy5-labeled PS beads for 10 min, fixed, stained with phalloidin, and observed by confocal microscopy. The intensities of F-actin across the targets were quantified using ImageJ. Distance 0 µm is the center of the PS bead. *N* = 37 beads for Ctrl and *n* = 6 beads for BAPTA-AM. **E** BMDMs treated with BAPTA-AM were incubated with Cy5- and pHrodo-labeled PS beads for 10 min and analyzed by flow cytometry. Data are mean ± s.e.m. from *n* = 3 independent experiments. NS, not significant; *P < 0.05; one-way ANOVA. **F**, **G** CellMask-stained BMDMs were treated with or without BAPTA-AM, incubated with Cy3-labeled PS beads, and observed by time-lapse confocal microscopy (**F**). The amount of time required for internalization was measured (**G**). The same colored arrowheads indicate the same PS bead being engulfed. Scale bar, 20 µm. Data are mean ± s.e.m. from *n* = 15 beads for Ctrl and *n* = 11 beads for BAPTA-AM. ***P < 0.001; two-tailed unpaired Student’s *t* test. **H**, **I** J774A.1 cells stably expressing GFP-CAAX were treated with or without BAPTA-AM, incubated with Cy3-labeled PS beads, and observed by time-lapse confocal microscopy (**H**). The amount of time required for internalization was measured (**I**). The time begins when the phagocytic cup forms at the interface between the asterisked PS beads and phagocytes. Scale bar, 10 µm. Data are mean ± s.e.m. from *n* = 8 beads for Ctrl and *n* = 5 beads for BAPTA-AM. **P < 0.01; two-tailed unpaired Student’s *t* test.

Depletion of extra- and intracellular calcium or inhibition of molecules involved in calcium flux abrogates engulfment of apoptotic cells [24–26, 28]. Recently, our group also reported that extracellular calcium is essential for efferocytosis because it is necessary for binding of PSRs and bridging molecules to PS and elevation of the intracellular calcium level [27]. Nevertheless, the role of intracellular calcium in phagocytes during efferocytosis remains unclear. To investigate this, we first confirmed the effect of intracellular calcium on engulfment of PS beads, 6–8 µm in diameter similar to the physiological apoptotic cell size and surrogates of apoptotic cells. We decreased intracellular calcium using BAPTA-AM, an intracellular calcium chelator, (Supplementary Fig. 1A) and evaluated its effects on engulfment of PS beads by bone marrow-derived macrophages (BMDMs). BAPTA-AM efficiently blocked elevation of the intracellular calcium level during engulfment of PS beads and inhibited engulfment of PS beads (Supplementary Fig. 1,B, C and video 1, and Fig. 1A). BAPTA-AM also impeded engulfment of apoptotic cells by BMDMs, as measured by the percentage and mean fluorescence intensity (MFI) of phagocytes engulfing apoptotic cells (Fig. 1B). The inhibitory effect of intracellular calcium depletion was not restricted to BMDMs but was also observed in primary macrophages and macrophage cell lines, peritoneal macrophages (PMs) and RAW264.7/J774A.1 cells, respectively (Supplementary Fig. 2A–D). These findings suggest that intracellular calcium affects general, not cell type-specific, engulfment machinery.

We thus conducted crucial experiments to determine the effect of intracellular calcium depletion on each step of efferocytosis [30]. Unexpectedly, the experiments failed to provide information about which specific step of efferocytosis is affected by intracellular calcium depletion. The number of PS beads or apoptotic cells binding to phagocytes was comparable between control and BAPTA-AM-treated BMDMs upon incubation at 4 °C (Fig. 1C and Supplementary Fig. 2E). F-actin formation and Rac1 activation, which were indicated by phalloidin staining and a Rac1 biosensor, respectively, and are necessary for internalization of the targets during efferocytosis [31], around PS beads were also indistinguishable between control and BAPTA-AM-treated phagocytes (Fig. 1D, and Supplementary Fig. 3A, B and video 2). Phagolysosomal acidification, which was assessed using the pH-sensitive dye pHrodo, was unaltered by BAPTA-AM treatment (Fig. 1E). We thus closely scrutinized the efferocytosis steps and complete internalization of targets in phagocytes treated with BAPTA-AM. We employed time-lapse confocal microscopy and measured the time required from phagocytic cup formation/extension at the interface between PS beads and phagocytes to phagocytic cup closure, where phagocytic cup closure indicates that the target is completely surrounded by the plasma membrane and pulled into phagocytes (Supplementary Fig. 3C and video 3). Intriguingly, phagocytes treated with BAPTA-AM than control phagocytes required more time for internalization of the targets. (Fig. 1F–I, and Supplementary video 4, 5). These findings suggest that intracellular calcium affects efferocytosis by modulating the internalization step of efferocytosis.

### Intracellular calcium is required for F-actin disassembly and MLC phosphorylation

**Fig. 2:**
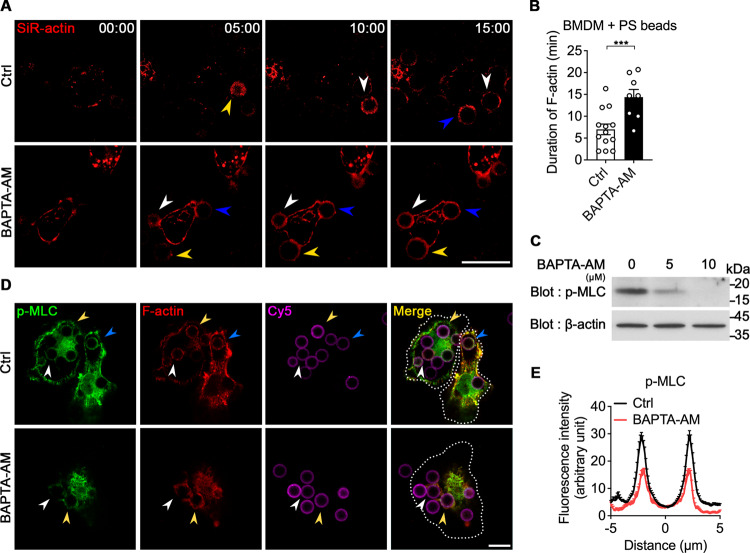
Intracellular calcium depletion affects F-actin disassembly and MLC phosphorylation during engulfment of PS beads. **A**, **B** BMDMs pretreated with BAPTA-AM were stained with SiR-actin, incubated with Cy3-labeled PS beads, and observed by time-lapse confocal microscopy (**A**). The duration of the F-actin signal was measured (**B**). The same colored arrowheads indicate the same PS bead. Scale bar, 20 µm. Data are mean ± s.e.m. from *n* = 13 beads for Ctrl and *n* = 8 beads for BAPTA-AM. ***P < 0.001; two-tailed unpaired Student’s *t* test. **C** BMDMs treated with the indicated concentration of BAPTA-AM were lysed, and proteins in the lysates were detected by the indicated antibodies using western blotting. **D**, **E** BMDMs pretreated with or without BAPTA-AM were incubated with Cy5-labeled PS beads for 10 min, fixed, stained with phalloidin and an anti-p-MLC antibody, and observed by confocal microscopy (**D**). The intensities of p-MLC were quantified using ImageJ (**E**). The dashed lines represent the cell boundaries and the same colored arrowheads indicate the same PS bead. Distance 0 µm is the center of the PS bead. Scale bar, 10 µm. *n* = 37 beads for Ctrl and *n* = 6 beads for BAPTA-AM.

The internalization step defect by intracellular calcium depletion can be caused by a defect in phagocytic cup extension and/or in phagocytic cup closure. Unfortunately, the real-time confocal microscopy which we used cannot efficiently distinguish the phagocytic cup closure defect from the phagocytic cup extension defect. However, we first hypothesized that the impaired internalization of PS beads was primarily attributed to a defect in phagocytic cup closure rather than its extension. This hypothesis was based on the observation that Rac1 activation and F-actin formation were comparable between control and BAPTA-AM treated phagocytes, although phagocytic cup extension was likely slowed down in phagocytes treated with BAPTA-AM (Fig. 1D, and Supplementary Fig. 3A, B and video 2). Phagocytic cup closure occurs concomitant with F-actin disassembly and MLC phosphorylation, which induces myosin II-mediated contraction [19, 21]. Due to the defect of phagocytic cup closure in intracellular calcium-depleted phagocytes, we next asked whether intracellular calcium depletion affects F-actin disassembly around the phagocytic cup during engulfment of PS beads. To this end, we stained BMDMs with SiR-actin, a live F-actin probe, and observed them by time-lapse confocal microscopy. F-actin signals around PS beads persisted longer in BAPTA-AM-treated BMDMs than in control BMDMs (Fig. 2A, B and Supplementary video 6), indicating that intracellular calcium depletion delays F-actin disassembly at the phagocytic cup during efferocytosis. Next, we tested whether intracellular calcium depletion also affects MLC phosphorylation, which is indicative of myosin II-mediated contraction. BAPTA-AM treatment diminished the basal level of phospho-MLC (p-MLC) in BMDMs (Fig. 2C), consistent with the previous observations [32]. In particular, BAPTA-AM treatment drastically lowered the level of p-MLC around PS beads (Fig. 2D, E). These data imply that intracellular calcium is at least necessary for F-actin disassembly and MLC phosphorylation leading to phagocytic cup closure.

### Intracellular calcium induces the association of Calmodulin with MLCK during efferocytosis

**Fig. 3:**
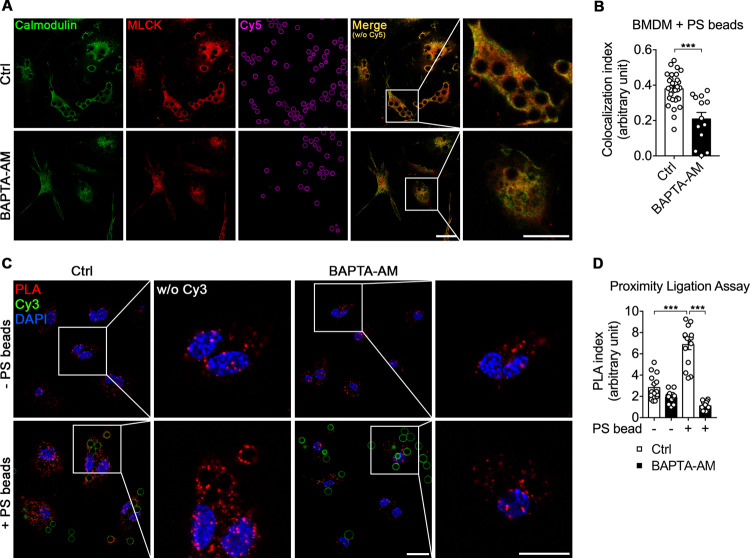
Intracellular calcium depletion weakens the association of Calmodulin with MLCK. **A**, **B** BMDMs pretreated with or without BAPTA-AM were incubated with Cy5-labeled PS beads for 10 min, fixed, stained with anti-Calmodulin and anti-MLCK antibodies, and observed by confocal microscopy (**A**). The colocalization index was calculated as the ratio of the fluorescence intensity overlap (**B**). Scale bar, 20 µm. Data are mean ± s.e.m. from *n* = 32 beads for Ctrl and *n* = 13 beads for BAPTA-AM. ***P < 0.001; two-tailed unpaired Student’s *t* test. **C**, **D** BMDMs pretreated with or without BAPTA-AM were incubated with Cy3-labeled PS beads for 10 min, fixed, blocked, incubated with anti-Calmodulin and anti-MLCK antibodies at 4 °C overnight, and incubated with the amplification solution. Images were acquired by confocal microscopy (**C**) and quantified (**D**). Scale bar, 20 µm. Data are mean ± s.e.m. from *n* = 15 randomly acquired images. ***P < 0.001; two-way ANOVA.

Intracellular calcium depletion by BAPTA-AM decreased the basal p-MLC level in phagocytes; therefore, in efferocytosis, intracellular calcium appears to be involved in a central MLC phosphorylation pathway in which calcium is necessary for binding of Calmodulin (CaM) to myosin light chain kinase (MLCK), which phosphorylates MLC [33]. To validate this, we compared the colocalization of CaM and MLCK between BAPTA-AM-treated and control BMDMs during engulfment of PS beads. When BMDMs were incubated with PS beads, the signals of CaM and MLCK colocalized around PS beads, but this colocalization was abrogated in BAPTA-AM-treated BMDMs (Fig. 3A, B). As an alternative approach to observe the association between CaM and MLCK, we used a proximity ligation assay (PLA) which enables to detect in situ protein interactions through protein-specific primary antibodies and their secondary antibodies with short sequence specific DNA strands, called PLA probes. It gives a fluorescent signal through rolling circle DNA synthesis when the PLA probes are in proximity, that is, when target proteins interact [34]. In the basal state, the PLA signal was detected ubiquitously in the form of dots in BMDMs. When BMDMs were incubated with PS beads, the number of PLA signals increased, and these signals were usually observed around PS beads. However, these signals were indistinguishable from those in control BMDMs upon treatment of BMDMs with BAPTA-AM (Fig. 3C, D). These data imply that intracellular calcium is required for the association of CaM with MLCK, which leads to MLCK activation and thus MLC phosphorylation, during efferocytosis.

### Inhibition of the CaM-MLCK-MLC axis delays internalization of the target

**Fig. 4:**
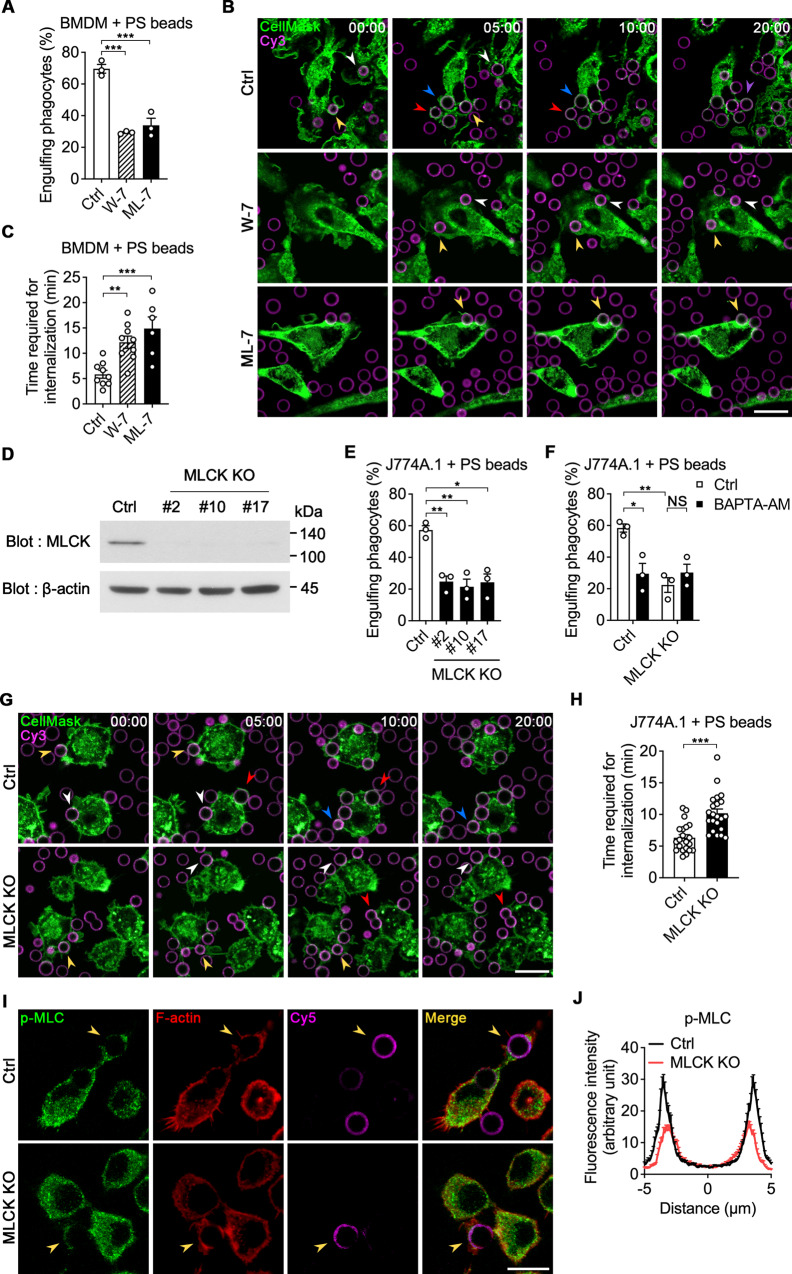
Intracellular calcium affects internalization of targets through the Calmodulin-MLCK axis. **A** BMDMs treated with the indicated inhibitors were incubated with Cy3-labeled PS beads for 15 min and analyzed by flow cytometry. Data are mean ± s.e.m. from *n* = 3 independent experiments. ***P < 0.001; one-way ANOVA. **B**, **C** CellMask-stained BMDMs treated with W-7 or ML-7 were incubated with Cy3-labeled PS beads and observed by time-lapse confocal microscopy (**B**). The amount of time required for internalization was measured (**C**). The same colored arrowheads indicate the same PS bead. Scale bar, 20 µm. Data are mean ± s.e.m. from *n* = 9 beads for Ctrl, *n* = 9 beads for W-7, and *n* = 6 beads for ML-7. **P < 0.01; ***P < 0.001; one-way ANOVA. **D** J774A.1 cells were transduced with MLCK-specific sgRNA and Cas9, and then puromycin-resistant cells were selected. MLCK expression in three candidate cell lines was observed by western blotting. **E** MLCK KO J774A.1 cells were incubated with Cy3-labeled PS beads for 15 min and analyzed by flow cytometry. Data are mean ± s.e.m. from *n* = 3 independent experiments. *P < 0.05; **P < 0.01; one-way ANOVA. **F** MLCK KO J774A.1 cells were treated with BAPTA-AM for 30 min and incubated with Cy3-labeled PS beads for 15 min. Engulfing phagocytes were analyzed by flow cytometry. Data are mean ± s.e.m. from *n* = 3 independent experiments. NS, not significant; *P < 0.05; **P < 0.01; two-way ANOVA. **G**, **H** MLCK KO J774A.1 cells stained with CellMask were incubated with Cy3-labeled PS beads and observed by time-lapse confocal microscopy (**G**). The amount of time required for internalization was measured (**H**). The same colored arrowheads indicate the same PS bead. Scale bar, 20 µm. Data are mean ± s.e.m. from *n* = 21 beads for Ctrl and *n* = 22 beads for MLCK KO. ***P < 0.001; two-tailed unpaired Student’s *t* test. **I**, **J** MLCK KO J774A.1 cells were incubated with Cy5-labeled PS beads for 10 min, fixed, stained with an anti-p-MLC antibody and phalloidin, and observed by confocal microscopy (**I**). The intensities of p-MLC around PS beads were quantified (**J**). Arrowheads indicate a PS bead being engulfed. Distance 0 µm indicates the center of the PS bead. Scale bar, 10 µm. *n* = 36 beads for Ctrl and *n* = 47 beads for MLCK KO.

We further validated the effect of the MLC phosphorylation pathway on phagocytic cup closure during efferocytosis. To this end, we used W-7 and ML-7, which block the binding of calcium to CaM and inhibit the kinase activity of MLCK, respectively. Both W-7 and ML-7 drastically inhibited engulfment of PS beads and apoptotic cells (Fig. 4A and Supplementary Fig. 4A). Next, we tested whether the effects of these inhibitors on efferocytosis are due to a defect of phagocytic cup closure. Upon incubation with PS beads, internalization of the targets took longer in BMDMs treated with the inhibitors than in control cells (Fig. 4B, C, and Supplementary video 7), suggesting that binding of calcium to CaM as well as the kinase activity of MLCK are required for phagocytic cup closure.

To more directly assess the effect of the CaM-MLCK-MLC axis on phagocytic cup closure during efferocytosis, we next generated MLCK-depleted J774A.1 stable cell lines (MLCK KO) using the CRISPR/Cas9 system (Fig. 4D). MLCK KO cells engulfed PS beads and apoptotic cells much less efficiently than control cells, as measured by the percentage of phagocytes engulfing targets (Fig. 4E and Supplementary Fig. 4B). However, engulfment of PS beads by MLCK KO cells was not further inhibited by intracellular calcium depletion (Fig. 4F), suggesting that intracellular calcium affects efferocytosis through the CaM-MLCK-MLC axis. We further investigated whether impaired efferocytosis by MLCK KO cells is caused by a defect of phagocytic cup closure. When engulfment of PS beads was observed by time-lapse microscopy, internalization of the targets took longer in MLCK KO cells than in control cells (Fig. 4G, H and Supplementary video 8). In addition, the level of p-MLC around PS beads was lower in MLCK KO cells than in control cells, whereas the phagolysosomal acidification, binding ability, and F-actin formation were comparable between control and MLCK KO cells during engulfment of PS beads (Fig. 4I, J and Supplementary Fig. 4C–E). Collectively, these data suggest that intracellular calcium activates the CaM-MLCK-MLC axis, leading to phagocytic cup closure during efferocytosis.

### Blockade of Mertk-mediated calcium influx delays internalization of the target

**Fig. 5:**
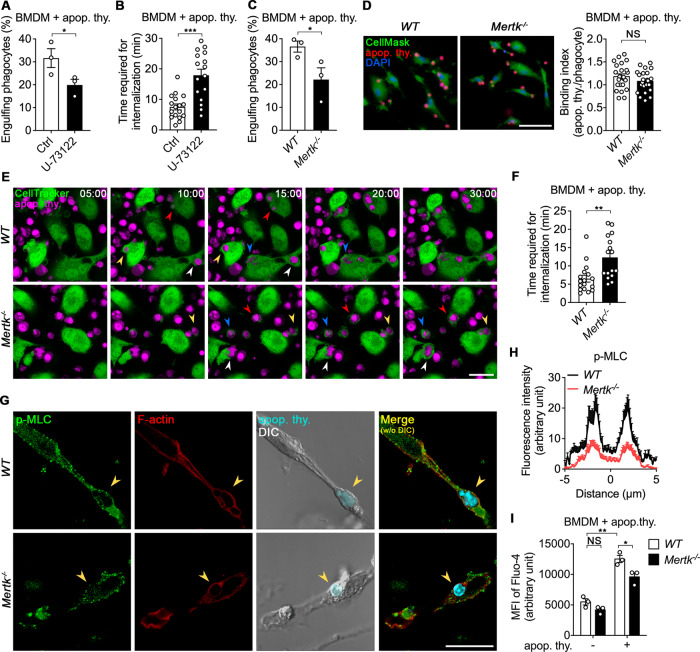
Interference with Mertk-mediated SOCE signaling delays internalization of apoptotic cells during efferocytosis. **A** BMDMs treated with U-73122 were incubated with pHrodo-stained apoptotic thymocytes for 30 min and analyzed by flow cytometry. Data are mean ± s.e.m. from *n* = 3 independent experiments. *P < 0.05; two-tailed paired Student’s *t* test. **B** BMDMs treated with U-73122 were incubated with TAMRA-stained apoptotic thymocytes for 1 h and observed by time-lapse confocal microscopy. The amount of time required for internalization was measured. Data are mean ± s.e.m. from *n* = 17 apoptotic cells for Ctrl and *n* = 16 apoptotic cells for U-73122. ***P < 0.001; two-tailed unpaired Student’s *t* test. **C** BMDMs derived from *WT* and *Mertk*^*−/−*^ mice were incubated with pHrodo-stained apoptotic cells for 30 min and analyzed by flow cytometry. Data are mean ± s.e.m. from *n* = 3 independent experiments. *P < 0.05; two-tailed paired Student’s *t* test. **D** *WT* and *Mertk*^*−/−*^ BMDMs stained with CellMask were incubated with TAMRA-stained apoptotic thymocytes at 4 °C for 2 h, fixed, stained with DAPI, and observed by fluorescence microscopy. The number of bound apoptotic cells per phagocyte was quantified. Data are mean ± s.e.m. from *n* = 23 randomly acquired images. NS, not significant; two-tailed unpaired Student’s *t* test. **E**, **F** *WT* and *Mertk*^*−/−*^ BMDMs stained with CellTracker were incubated with TAMRA-stained apoptotic thymocytes for 1 h and observed by time-lapse confocal microscopy (**E**). The amount of time required for internalization was measured (**F**). The same colored arrowheads indicate the same apoptotic cell. Scale bar, 20 µm. Data are mean ± s.e.m. from *n* = 17 apoptotic cells for *WT* and *n* = 16 apoptotic cells for *Mertk*^*−/−*^. **P < 0.01; two-tailed unpaired Student’s *t* test. **G**, **H** *WT* and *Mertk*^*−/−*^ BMDMs were incubated with Hoechst 33342-stained apoptotic cells for 20 min, fixed, stained with phalloidin and an anti-p-MLC antibody, and observed by confocal microscopy (**G**). The intensities of p-MLC across the targets were measured using ImageJ (**H**). Scale bar, 20 µm. Data are mean ± s.e.m. from *n* = 22 apoptotic cells for *WT* and *n* = 16 apoptotic cells for *Mertk*^*−/−*^. **I**. *WT* and *Mertk*^*−/−*^ BMDMs stained with Fluo-4 were incubated with apoptotic cells for 5 min and analyzed by flow cytometry. Data are mean ± s.e.m. from *n* = 3 independent experiments. NS, not significant; *P < 0.05; **P < 0.01; two-way ANOVA.

Our group previously reported that SOCE is provoked by the Mertk-PLCγ1-IP_3_R axis upon apoptotic cell stimulation during efferocytosis, resulting in intracellular calcium elevation, and that a CRAC channel inhibitor delays phagocytic cup closure [26, 27]. These findings imply that intracellular calcium elevation during efferocytosis is important for phagocytic cup closure, and that disruption of the Mertk-PLCγ1-IP_3_R axis may perturb phagocytic cup closure. To validate this possibility, we first pharmacologically inhibited the Mertk-PLCγ1-IP_3_R axis. We treated BMDMs with U-73122, a PLCγ1 inhibitor, and measured efferocytosis. Efferocytosis was prominently diminished in BMDMs treated with U-73122 compared with control cells and internalization of apoptotic cells also took longer in BMDMs treated with U-73122 than in control cells (Fig. 5A, B), suggesting that the impairment of efferocytosis appeared to be due to defective phagocytic cup closure.

We also used BMDMs derived from *Mertk*^*−/−*^ mice to validate the effect of the axis on phagocytic cup closure. As reported previously [35], efferocytosis by *Mertk*^*−/−*^ BMDMs was inefficient compared with that by wild-type (*WT*) BMDMs (Fig. 5C). Interestingly, however, the inefficient efferocytosis by *Mertk*^*−/−*^ BMDMs did not appear to be solely due to the impaired binding capacity of phagocytes. The number of bound apoptotic cells per phagocyte was comparable between *WT* and *Mertk*^*−/−*^ BMDMs (Fig. 5D). Indeed, it has been reported that *WT* and *Mertk*^*−/−*^ peritoneal macrophages have comparable binding capacities and Mertk expression in phagocytes where TAM receptors are depleted fails to promote efferocytosis [14, 36]. These findings imply that another defect, in addition to the binding defect, is responsible for the inefficient efferocytosis by *Mertk*^*−/−*^ BMDMs, i.e., phagocytic cup closure may be impaired in *Mertk*^*−/−*^ BMDMs. To test this, the level of MLC phosphorylation and amount of time required for phagocytic cup closure were compared between *WT* and *Mertk*^*−/−*^ BMDMs during efferocytosis. Internalization of apoptotic cells was delayed in *Mertk*^*−/−*^ BMDMs compared with *WT* BMDMs and the level of MLC phosphorylation around targets was also lower in *Mertk*^*−/−*^ BMDMs than in *WT* BMDMs (Fig. 5E-H and Supplementary video 9). However, Rac1 activation and F-actin formation in *WT* and *Mertk*^*−/−*^ BMDMs were marginally different during efferocytosis (Supplementary Fig. 5A, B). In addition, the calcium level in *Mertk*^*−/−*^ BMDMs was lower than in *WT* BMDMs upon apoptotic cell stimulation (Fig. 5I). Collectively, these data suggest that a defect of phagocytic cup closure caused by lower elevation of the calcium level in *Mertk*^*−/−*^ BMDMs during efferocytosis contributes to inefficient engulfment of apoptotic cells and ultimately Mertk-mediated intracellular calcium elevation during efferocytosis activates the CaM-MLCK-MLC axis, leading to phagocytic cup closure.

## Discussion

We previously reported that PS on apoptotic cells induces SOCE though the Mertk-PLCγ1-IP_3_R pathway [27]. Mertk is an engulfment receptor that indirectly recognizes PS on apoptotic cells through Gas6 or ProS [36–38]. BMDMs derived from *Mertk*^*−/−*^ mice contain less phosphorylated PLCγ1 and IP_3_R than *WT* BMDMs, leading to inefficient SOCE upon apoptotic cell stimulation. Impairment of an engulfment receptor usually perturbs binding of phagocytes to apoptotic cells because engulfment receptors on phagocytes recognize and bind to apoptotic cells. However, the binding capacity of *Mertk*^*−/−*^ BMDMs did not appear to be markedly affected. The binding index of *Mertk*^*−/−*^ BMDMs was comparable with that of *WT* BMDMs (Fig. 5D). Other groups also reported that the binding defect is smaller than the efferocytosis defect in *Mertk*^*−/−*^ macrophages, and that *Mertk*^*−/−*^ macrophages exhibit a defect in internalization of apoptotic cells [14]. Nevertheless, efferocytosis by *Mertk*^*−/−*^ BMDMs was prominently lower than that by *WT* BMDMs in this study. This may be because other engulfment receptors such as Tim-4 can compensate for the ability of Mertk to bind to apoptotic cells, and that the major role of Mertk is in a process other than recognition of apoptotic cells in efferocytosis. Indeed, Mertk is not involved in recognition of apoptotic cells in certain types of macrophages where Tim-4 is expressed [36]. Thus, one important role of Mertk signaling is likely induction of SOCE, leading to phagocytic cup closure and internalization of apoptotic cells during efferocytosis. The roles of Mertk and Tim-4 in internalization and recognition of apoptotic cells, respectively, during efferocytosis seem to be functionally distinct.

Calcium flux is modulated during efferocytosis, and calcium both outside and inside of cells is indispensable for efficient efferocytosis [25, 26, 28]. In this study, we report that intracellular calcium is required for phagocytic cup closure leading to internalization of apoptotic cells. Intracellular calcium depletion induced stable F-actin assembly and decreased MLC phosphorylation at the phagocytic cup surrounding targets. Thus, intracellular calcium seems to be essential for both F-actin disassembly and MLC phosphorylation at the phagocytic cup. We also delineated a molecular mechanism by which intracellular calcium regulates MLC phosphorylation: intracellular calcium elevation during efferocytosis increases binding of calcium to CaM, which increases binding of CaM to MLCK, leading to MLCK activation and thus MLC phosphorylation. However, we did not investigate how intracellular calcium depletion stabilizes F-actin surrounding targets during efferocytosis. Nevertheless, calcium is indispensable for F-actin dynamics. In particular, a rapid increase of intracellular calcium such as that mediated by SOCE triggers changes in actin organization, called calcium-mediated actin reset, with calcium influx simultaneously leading to deconstruction of large parts of the actin cortex and formation of actin filaments near the center of the cell [39, 40]. In addition, intracellular calcium elevation promotes F-actin disassembly in various cell types and cellular processes [41, 42]. Likewise, it is feasible that rapid intracellular calcium elevation mediated by SOCE during efferocytosis causes F-actin disassembly, which is a prerequisite for phagocytic cup closure.

Intracellular calcium may be involved in steps of efferocytosis other than phagocytic cup closure. Especially in this study, the real-time confocal microscopy we used lacks sufficient resolution to differentiate between phagocytic cup extension and phagocytic cup closure. As a result, real-time confocal microscopy does not provide definitive evidence to solely attribute the defect to impaired phagocytic cup closure. Indeed, phagocytic cup extension seemed to be slowed down when intracellular calcium was depleted. Phagocytic cup extension is known to be facilitated by actin polymerization induced by Rac1 activation during efferocytosis. However, we discovered that F-actin formation and Rac1 activation were indistinguishable between control and calcium-depleted phagocytes. Therefore, it is possible that intracellular calcium might be involved in phagocytic cup extension through an unknown mechanism during efferocytosis, making it an intriguing topic for future research. In addition, Drp-1-mediated mitochondrial fission during efferocytosis enables continuous engulfment of apoptotic cells by facilitating vesicular trafficking and phagolysosomal degradation [28]. We also found that intracellular calcium depletion did not alter phagolysosomal acidification but it seemed to affect phagolysosomal degradation. Phagocytes engulfing TAMRA-stained apoptotic cells had a higher TAMRA MFI upon BAPTA-AM treatment than control phagocytes (data not shown). These suggest that phagolysosomal degradation is defective in the absence of intracellular calcium. Moreover, this observation is consistent with a recent report that macrophages lacking Trpm7, a calcium-conducting ion channel, fail to digest engulfed apoptotic cells [43]. Because intracellular calcium affect lysosomal calcium signaling and homeostasis [44] and phagolysosomal acidification was not affected by intracellular calcium depletion (Fig. 1E), intracellular calcium may be also involved in direct activation of lysosomal enzymes in efferocytosis. However, a further study is required to elucidate the molecular mechanism by which intracellular calcium regulates degradation of phagolysosomal cargoes.

In summary, intracellular calcium is elevated during efferocytosis, but the reason why has not been completely explored. In this study, we report that Mertk-mediated intracellular calcium elevation during efferocytosis is necessary for MLC phosphorylation and F-actin disassembly, leading to phagocytic cup closure and eventually internalization of the target. Efferocytosis is involved in resolution of inflammation, and defective efferocytosis is linked to autoimmunity; therefore, our observations may provide a new approach for immunoregulation.

## Materials and methods

### Cell culture and transfection

J774A.1, RAW264.7, and 293 T cells were cultured in Dulbecco’s Modified Eagle Medium (DMEM) containing 10% fetal bovine serum (FBS) and 1% penicillin-streptomycin-glutamine (PSQ). Primary mouse BMDMs were differentiated from bone marrow cells of C57BL/6 mice by culture in Roswell Park Memorial Institute (RPMI) 1640 medium supplemented with 20% L929-conditioned medium, 10% FBS, and 1% PSQ for 7 d. To obtain PMs, mouse exudate cells were collected after injection of cold phosphate-buffered saline (PBS) into the peritoneum. At 6 h after plating on nontreated culture dishes, floating cells were removed, and attached cells were used as resident PMs. PMs were maintained in RPMI 1640 medium containing 10% FBS and 1% PSQ. J774A.1 cells were transfected with Raichu-Rac1 (Raichu-Rac1 1011x) or GFP-CAAX (membrane-targeted GFP) using FuGENE according to the manufacturer’s protocol.

### Mice

C57BL/6 mice were purchased from Taconic Biosciences (Germantown, NY, USA). *Mertk*^−*/−*^ mice were purchased from Jackson Laboratory (Bar Harbor, ME, USA). Approximately 8–10-week-old mice were used to obtain BMDMs or PMs, and 4–6-week-old mice were used to generate apoptotic thymocytes regardless of sex. All mice were bred in a fully equipped animal facility with proper temperature and humidity. All experiments using mice were approved by the animal care and ethics committees of the Gwangju Institute of Science and Technology in accordance with the National Institute of Health Guide for the Care and Use of Laboratory Animals.

### Reagents

The inhibitors and other reagents used in this study were BAPTA-AM (B6769; Invitrogen, Carlsbad, CA, USA), ML-7 (I2764; Sigma-Aldrich, St. Louis, MO, USA), W-7 (681629, Sigma-Aldrich), U-73122 (662035; Calbiochem, Darmstadt, Germany), TAMRA-SE (C1171, Invitrogen), pHrodo Red-SE (P36600, Invitrogen), CellTracker Green CMFDA Dye (C7025, Invitrogen), CellMask Green Plasma Membrane Stain (C37608, Invitrogen), SiR-actin kit (CY-SC001; Cytoskeleton, Denver, CO, USA), Alexa Fluor 594-conjugated phalloidin (A12381, Invitrogen), Hoechst 33342 (H1399, Invitrogen), Duolink Proximity Ligation Assay Kit (DUO92101, Sigma-Aldrich), dexamethasone (D1756, Sigma-Aldrich), NeutrAvidin-coated polystyrene particles (NVP-60–5; Spherotech, Lake Forest, IL, USA), FuGENE HD Transfection Reagent (E2311; Promega, Madison, WI, USA), Fluo-4 AM (F14201; Thermo Fisher Scientific, Waltham, MA, USA), Pluronic F-127 (P3000MP, Invitrogen), and Rac1 Activation Assay Kit (17–283; Merck, Darmstadt, Germany). The antibodies used in this study were anti-pS20 MLC (ab2480; Abcam, Cambridge, UK), anti-pT18/pS19 MLC (3674 S; Cell Signaling Technology, Danvers, MA, USA), anti-MLCK (ab76092, Abcam), anti-MLCK (M7905, Sigma-Aldrich), anti-Calmodulin (05–173, Sigma-Aldrich), and anti-β-actin (SC47778; Santa Cruz Biotechnology, Dallas, TX, USA). Alexa Fluor 488-conjugated anti-mouse, Alexa Fluor 488-conjugated anti-rabbit, and Alexa Fluor 594-conjugated anti-rabbit antibodies (A11029, A11008, and A11037) were purchased from Invitrogen. The Raichu-Rac1 1011x plasmid was a generous gift provided by Michiyuki Matsuda at Kyoto University. The CAAX (Cys-Val-Ile-Met) sequence of GFP-CAAX was confirmed introduced in pEGFP-N1 vector.

### Binding assay

BMDMs or J774A.1 cells were plated on cover glasses with a diameter of 18 mm in a 12-well nontreated culture plate. At 1 d after plating, the cells were stained with CellMask in PBS and treated with 5–10 μM BAPTA-AM in calcium-free buffer (10 mM HEPES, 150 mM NaCl, 5 mM KCl, 0.1% glucose, 1% FBS, and 2.5 mM probenecid, pH 7.4). Then, cells were incubated with Cy3-labeled PS beads or TAMRA-stained apoptotic thymocytes at 4 °C for 2 h. To synthesize PS beads, 6.0–8.0 μm NeutrAvidin-coated polystyrene particles were coated with biotin-PS. Cy3- and biotin-conjugated 21-mer dsDNAs were used to label the PS beads. Apoptotic thymocytes were generated by incubating cells with 1 μM dexamethasone at 37 °C for 16 h. The cells were washed with PBS, fixed in 4% paraformaldehyde, and stained with Hoechst 33342. Images were acquired by fluorescence microscopy (Axio Imager D2; Zeiss, Jena, Germany).

### Phagocytosis assay

The phagocytosis assay was performed as described previously [26]. Briefly, BMDMs, PMs, RAW264.7 cells, or J774A.1 cells were plated in a 24-well plate. The day after plating, phagocytes were treated with 10 μM BAPTA-AM, 2.5 μM W-7, 25 μM ML-7, or 1 μM U-73122. To chelate intracellular calcium, cells were pretreated with BAPTA-AM in calcium-free buffer for 30 min before treatment with engulfment targets and W-7, ML-7, or U-73122 was cotreated with engulfment targets. Cells were incubated with engulfment targets (Cy3-labeled PS beads, TAMRA-stained apoptotic thymocytes, or pHrodo-stained apoptotic thymocytes) at 37 °C for the indicated durations. For acidification test, the day after plating, BMDMs were pretreated with 5 μM BAPTA-AM or J774A.1 cells were pretreated with 10 μM BAPTA-AM in calcium-free buffer for 30 min at 37 °C. Cy5- and biotin-conjugated 21-mer dsDNAs were used to label the PS beads. Then, pHrodo, a pH-sensitive dye, was used to stain Cy5-labeled PS beads. Cells were incubated with Cy5-labeled PS beads or pHrodo-stained Cy5-labeled PS beads at 37 °C for the indicated durations. Phagocytes were washed with ice-cold PBS to remove unbound targets, detached, and analyzed by flow cytometry (BD FACS Canto II; BD Biosciences, San Jose, CA, USA) using Flowjo software (FlowJo LLC, Ashland, OR, USA).

### Immunofluorescence microscopy

BMDMs or J774A.1 cells plated on cover glasses with a diameter of 18 mm in a 12-well nontreated culture plate were incubated with targets (Cy3-labeled PS beads, Cy5-labeled PS beads, or Hoechst 33342-stained apoptotic thymocytes) at 37 °C for the desired durations. Cells were pretreated with 10 μM BAPTA-AM in calcium-free buffer for 30 min before treatment with engulfment targets. Cells were fixed with 4% paraformaldehyde in PBS, permeabilized with 0.1% Triton X-100, and blocked with 3% bovine serum albumin. Then, cells were stained with an anti-p-MLC antibody and Alexa Fluor 594-conjugated phalloidin at 4 °C overnight. After washing with PBS, cells were stained with an Alexa Fluor 488-conjugated anti-rabbit secondary antibody. To observe colocalization of MLCK and Calmodulin, staining was performed using anti-MLCK and anti-Calmodulin primary antibodies, followed by Alexa Fluor 488-conjugated anti-mouse and Alexa Fluor 594-conjugated anti-rabbit secondary antibodies. Images were acquired by confocal microscopy (FV1000 and FV3000; Olympus, Tokyo, Japan).

### Time-lapse confocal microscopy

BMDMs or J774A.1 cells plated on 35 mm confocal dishes were stained with CellTracker or CellMask at 37 °C for 30 min. Cells were labeled with 5 μM SiR-actin and 10 μM verapamil together with the cell staining dye to observe F-actin formation. Cells were pretreated with 5 μM BAPTA-AM and the staining dye for 30 min. Cells were cotreated with 2.5 μM W-7, 25 μM ML-7, or 1 μM U-73122 and the engulfment targets. After adding the desired targets, the cells were immediately observed by confocal microscopy. Images were taken every 20 s for 30 min or 10 s for 1 h at 37 °C with 5% CO_2_ using confocal microscopes (FV1000 and FV3000). For FRET analysis, Raichu-Rac1 expressing J774A.1 cells were incubated with or without 5 μM BAPTA-AM for 30 min. Then, PS beads were added and the cells were immediately observed by confocal microscopy every 5 s for 20 min (FV3000).

### Detection of the intracellular calcium level

BMDMs were plated in a 24-well plate. The day after plating, cells were pretreated with 1 μM Fluo-4, final concentration 0.04% F-127, and with or without 10 μM BAPTA-AM in calcium-free buffer for 30 min at 37 °C. The cells were washed with ice-cold PBS to remove any remaining reagents, detached, and analyzed by flow cytometry. For live cell imaging of intracellular calcium, BMDMs plated on a 35 mm confocal dish were pretreated with 1 μM Fluo-4, final concentration 0.04% F-127, and with or without 5 μM BAPTA-AM in calcium-free buffer for 30 min at 37 °C. The cells were then washed with calcium-free buffer and observed by confocal microscopy equipped with a resonant scanner. After one minute, PS beads were added, and images were taken continuously every second for 10 min (FV3000).

### Proximity ligation assay

The proximity ligation assay was performed as described previously [15]. BMDMs plated on cover glasses with a diameter of 18 mm in a 12-well nontreated culture plate were incubated with 10 μM BAPTA-AM for 30 min. After adding Cy3-labeled PS beads, phagocytes were fixed with 4% paraformaldehyde, blocked with blocking solution, and incubated with anti-MLCK and anti-Calmodulin antibodies at 4 °C overnight. For ligation and amplification, cells were incubated with anti-rabbit and anti-mouse PLA probes and fixed with Duolink mounting media with DAPI. Images were acquired using a confocal microscope (FV1000).

### Generation of MLCK-depleted cell lines using the CRISPR/Cas9 system

293 T cells were seeded in a 12-well plate and transfected with packaging plasmids (psPAX2 and pMD2.G) and pCMV-Cas9-puromycin plasmids containing sgRNA for *Mylk* (gene encoding MLCK) or nontargeting control sgRNA. FuGENE was used for transient transfection according to the manufacturer’s protocol. The sequence of sgRNA targeting Exon14 of *Mylk* was 5’-TCATACTGGCCGGCATGCCA-3’. At 24 h post-transfection, the medium was changed to DMEM containing 30% FBS. After 24 and 48 h, lentiviral particles were collected by centrifugation and purified using a filter with a pore size of 0.45 μm. Then, J774A.1 cells were transduced with lentiviral vectors or empty vector controls. For 2 weeks, medium was replaced every 2–3 d with selection medium containing puromycin until nontransduced cells were killed. MLCK KO and control cells were plated at very low densities, and colonies formed by single cells were expanded in separate wells of a 24-well plate. Target gene expression was confirmed by western blotting.

### Immunoblotting

BMDMs or J774A.1 cells were lysed using lysis buffer (10 μg/mL AEBSF, 10 μg/mL aprotinin, 10 μg/mL pepstatin, 10 μg/mL leupeptin, 150 mM NaCl, 10 mM NaF, 10 mM NaPP, 1 mM Na_3_VO_4_, 1% Triton X-100, and 50 mM Tris (pH 7.6)). The lysates were separated by SDS-PAGE and transferred onto a nitrocellulose membrane. Proteins were detected by immunoblotting.

### Rac1 activation pulldown assay

BMDMs were incubated with apoptotic thymocytes for 20 min at 37 °C, followed by removal of unbound targets by washing with ice-cold PBS. The cells were lysed using lysis buffer and the Rac1 pulldown assay was performed according to the manufacturer’s protocol for the Rac1 Activation Assay Kit. In this assay, the GTP-bound active form of Rac1 specifically bound to the GST-tagged PAK-1 PBD fusion protein conjugated to glutathione agarose beads. The active Rac1 was eluted from the beads and analyzed by western blotting using an anti-Rac1 antibody (05–389, Merck).

### FRET analysis

In this study, two types of images were obtained, donor channel image (CFP) and acceptor channel image (YFP), from J774A.1 cells expressing Raichu-Rac1. We employed an image analysis pipeline which consists of several steps, including image preprocessing, FRET ratio calculation, edge detection, and image enhancement using Python. First, donor and acceptor images were thresholded to remove background noise and enhance the signal-to-noise ratio. The threshold values were determined using Otsu’s method. Next, FRET ratio was calculated by dividing the acceptor intensity by the donor intensity. The resulting FRET ratio image was normalized and prepared for further processing. Subsequently, Canny edge detection was applied and the detected edges were dilated followed by Gaussian blurring. The blurred edges were then used as a mask to combine with the original FRET ratio image, creating a composite image. To improve visualization, a custom colormap was applied to the FRET ratio image, and morphological closing operations were performed using a 5×5 pixel kernel. The processed image was then blurred using a 7×7 pixel Gaussian filter. An alpha channel was added to the processed FRET ratio image to control its transparency. The opacity-adjusted FRET image was merged with a Differential Interference Contrast (DIC) microscopy image. A mask was created based on the alpha channel of the opacity-adjusted FRET image and then converted to the RGB color space if needed. Using the mask, the opacity-adjusted FRET image and the DIC image were blended with a specified weight (0.5). This image analysis pipeline facilitated efficient processing, calculation, and presentation of FRET data in a research setting. By analyzing the FRET ratio, we could determine the level of Rac1 activity in a given cell.

### Statistical analysis

Data are shown as the mean ± standard error of mean (s.e.m.). To analyze statistical differences, the Student’s two-tailed *t* test was applied for experiments involving two groups. A one- or two-way analysis of variance (ANOVA) was performed for experiments involving three or more groups using GraphPad Prism 9 software (Prism 9, GraphPad Software, La Jolla, CA, USA). Significance was defined when *p*-values were less than 0.05.

## Supplementary information

Supplementary video 1

Supplementary video 2

Supplementary video 3

Supplementary video 4

Supplementary video 5

Supplementary video 6

Supplementary video 7

Supplementary video 8

Supplementary video 9

Supplementary figure 1

Supplementary figure 2

Supplementary figure 3

Supplementary figure 4

Supplementary figure 5

Supplementary figure legends

Supplementary original WB

Reproducibility checklist

## Data Availability

Data from this study are available within the main text and the supplementary information or from the corresponding authors (D.P.) upon reasonable request.
