# Supplementary figures and images for: Internalization of apoptotic cells during efferocytosis requires Mertk-mediated calcium influx
Source: Cell Death Dis. 2023 Jun 30;14(6):391. doi: 10.1038/s41419-023-05925-7 (PMC10313764; doi:10.1038/s41419-023-05925-7)

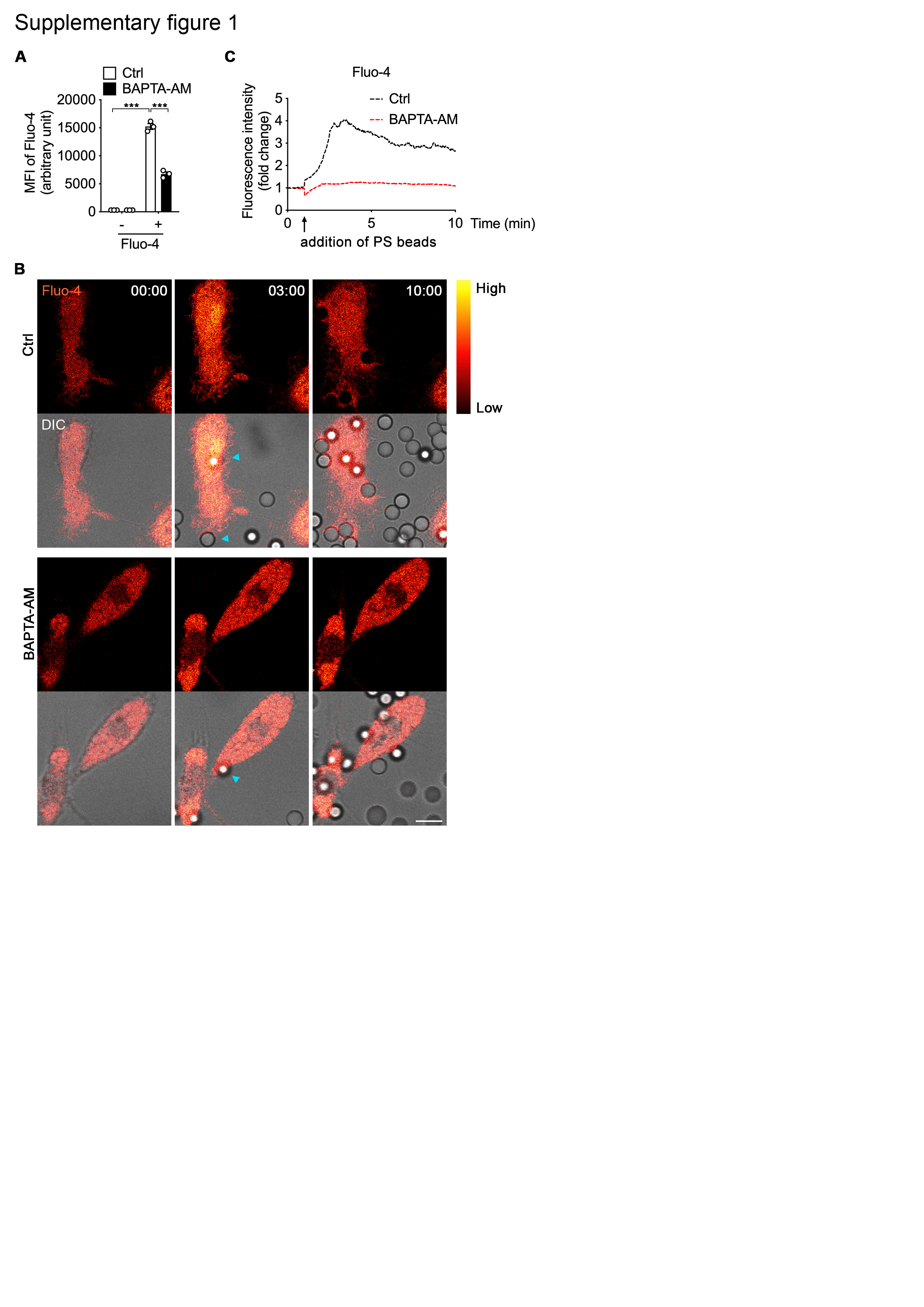

Supplement: Supplementary file 10 — Supplementary figure 1 [file 41419_2023_5925_MOESM10_ESM.tif]

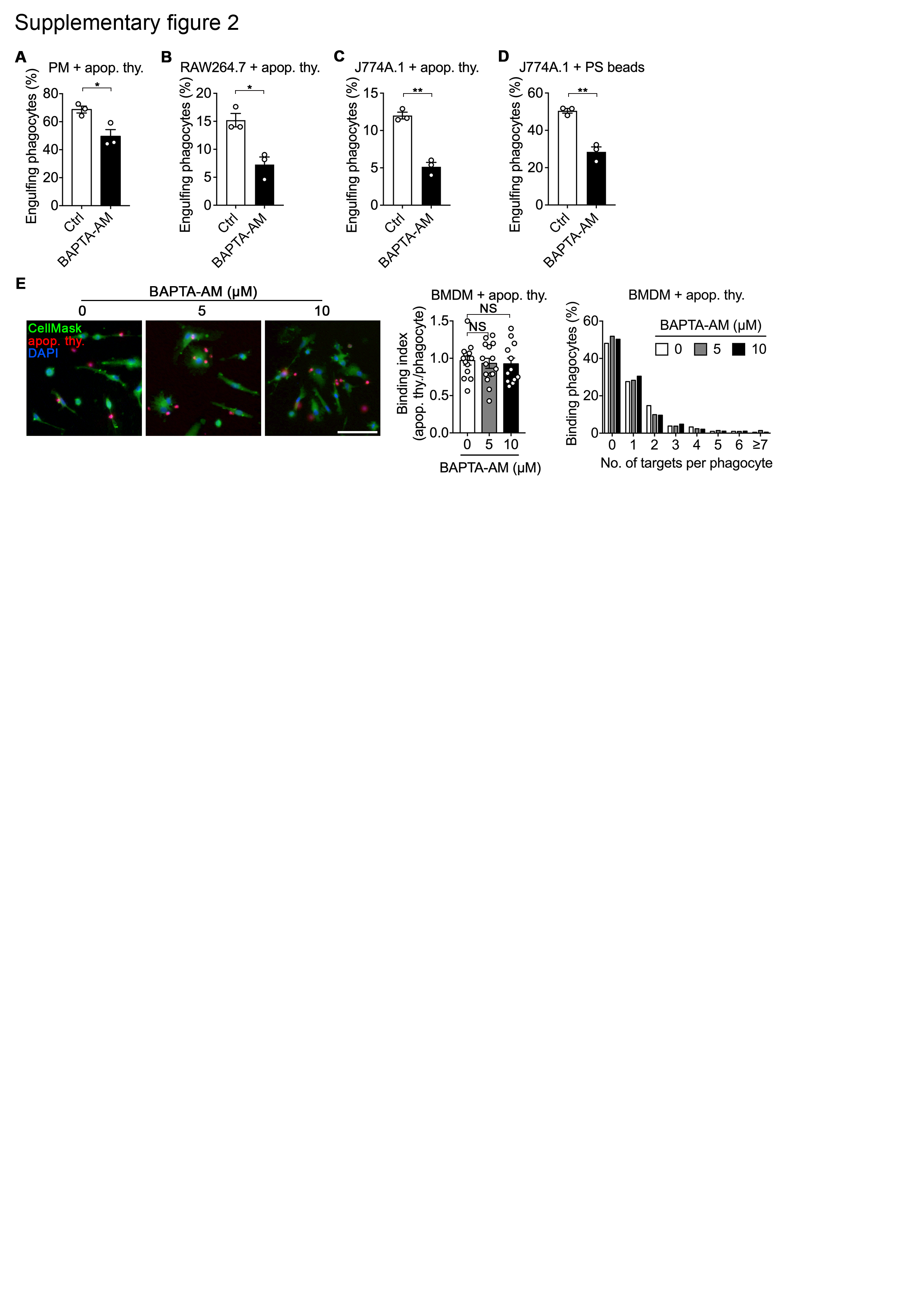

Supplement: Supplementary file 11 — Supplementary figure 2 [file 41419_2023_5925_MOESM11_ESM.tif]

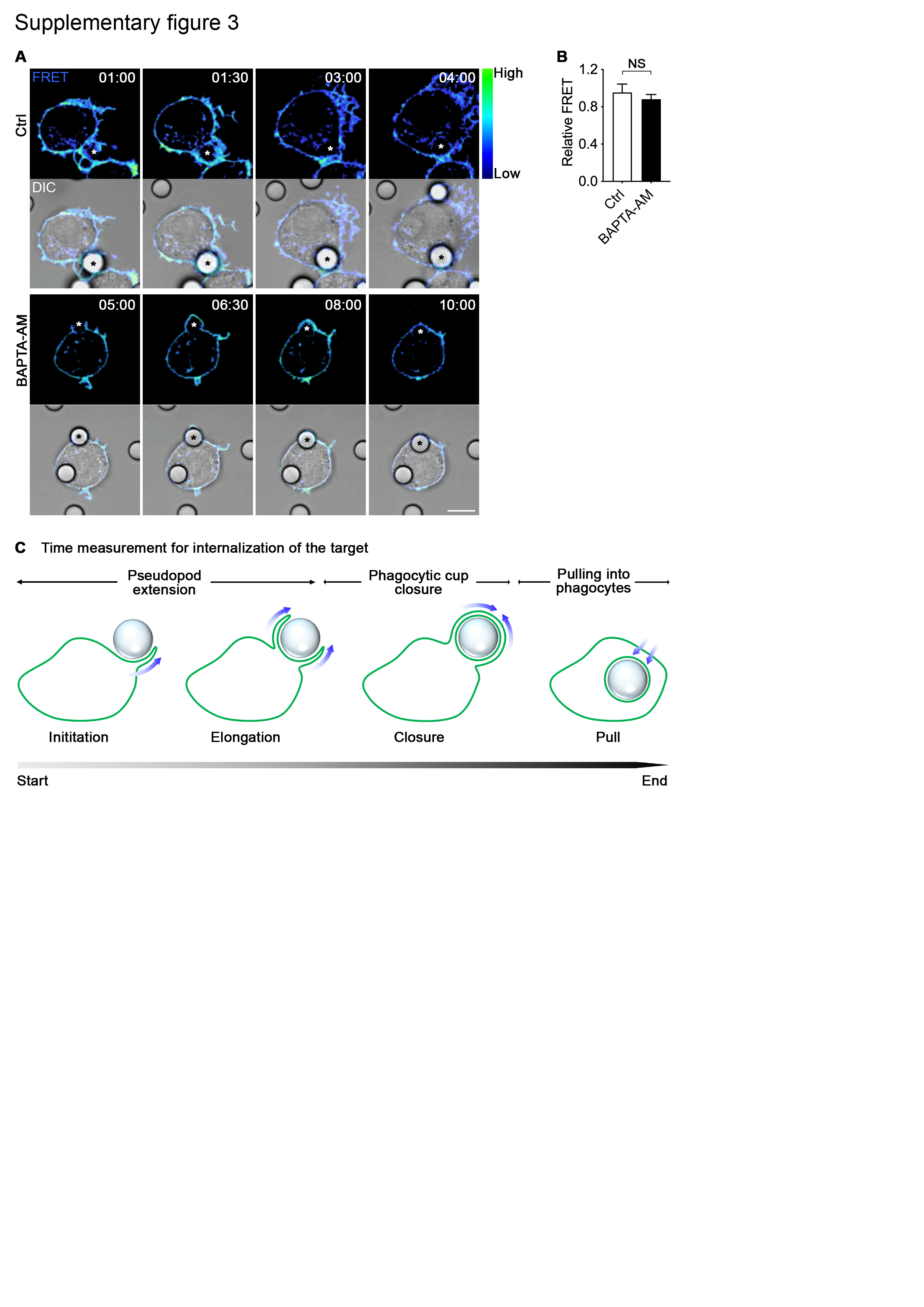

Supplement: Supplementary file 12 — Supplementary figure 3 [file 41419_2023_5925_MOESM12_ESM.tif]

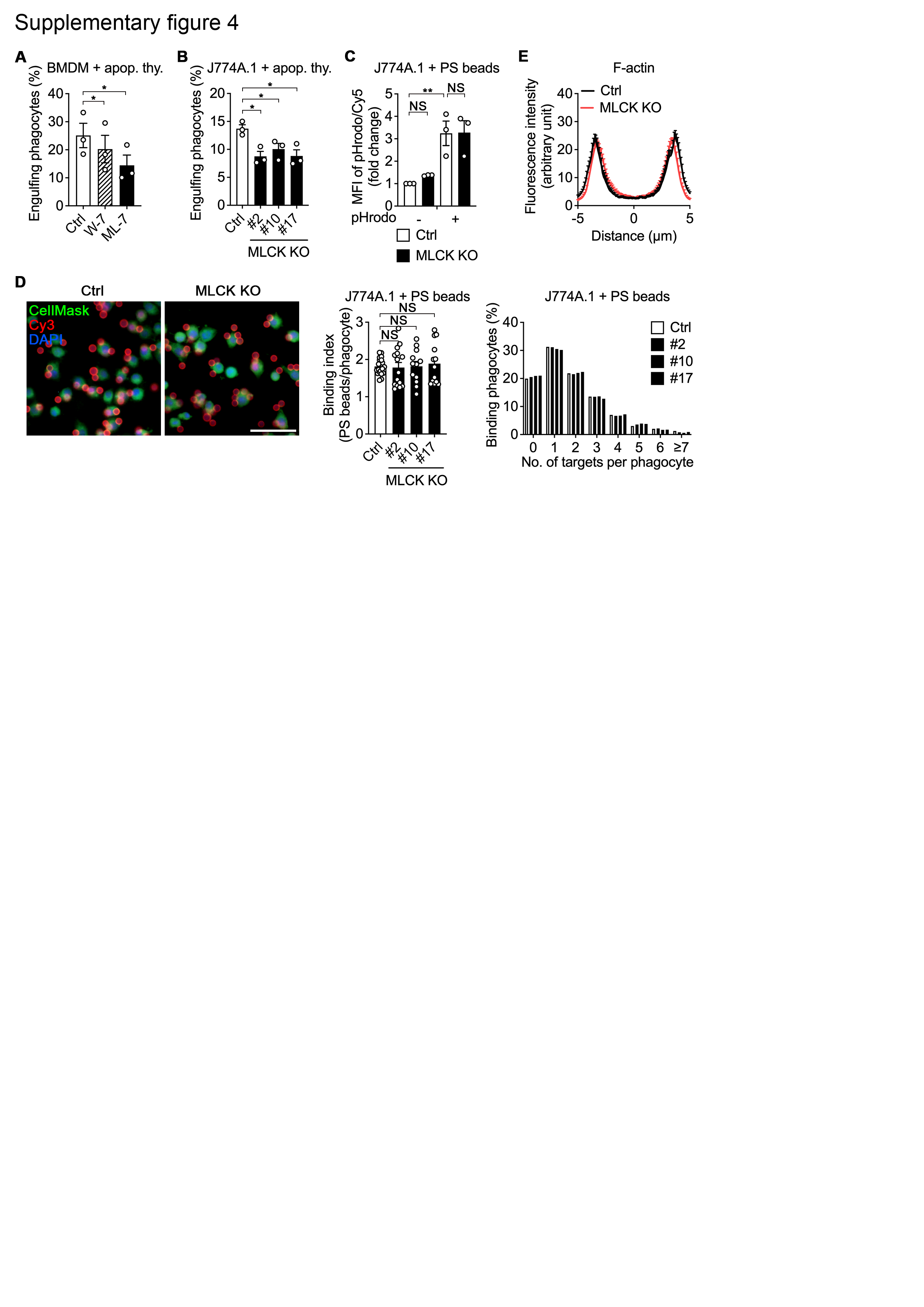

Supplement: Supplementary file 13 — Supplementary figure 4 [file 41419_2023_5925_MOESM13_ESM.tif]

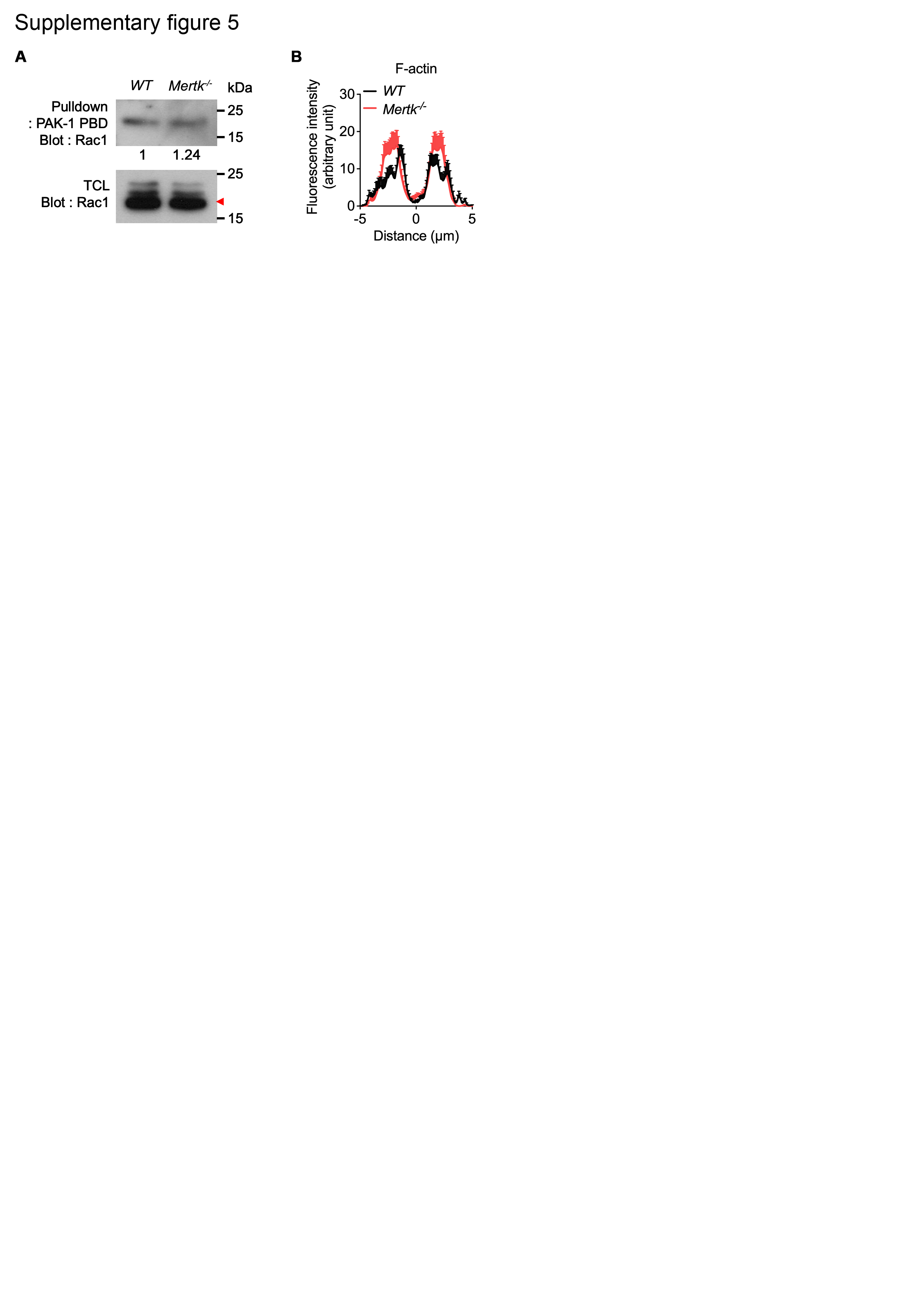

Supplement: Supplementary file 14 — Supplementary figure 5 [file 41419_2023_5925_MOESM14_ESM.tif]

Figure 2

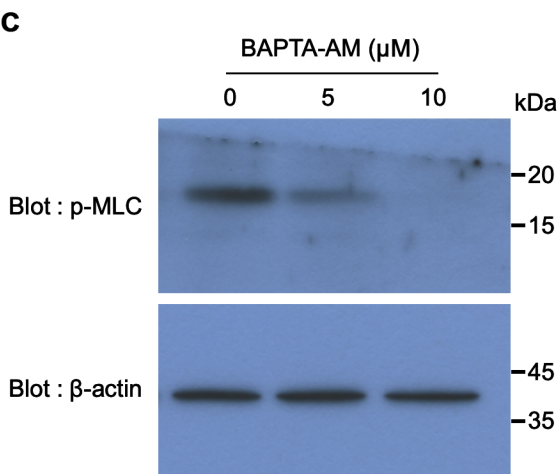

Figure 4

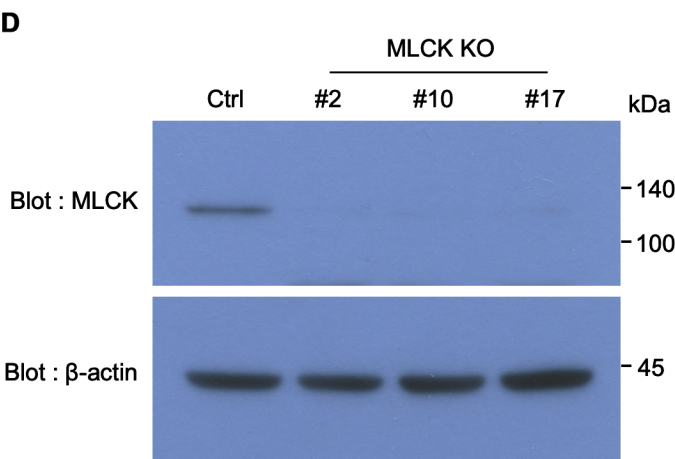

Supplementary figure 5

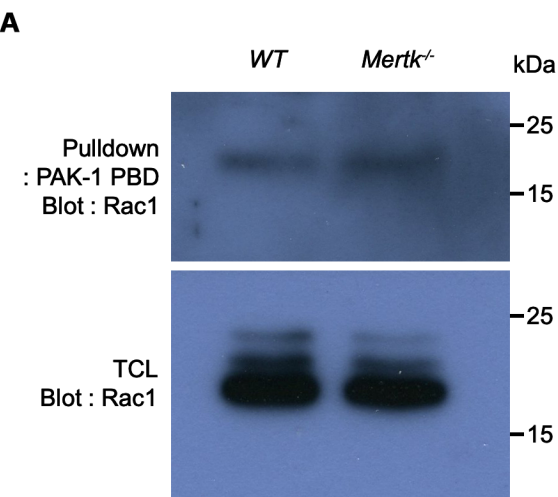

Supplement: Supplementary file 16 — Supplementary original WB [file 41419_2023_5925_MOESM16_ESM.pdf]
