# Supplementary material for: Internalization of apoptotic cells during efferocytosis requires Mertk-mediated calcium influx
Source: Cell Death Dis. 2023 Jun 30;14(6):391. doi: 10.1038/s41419-023-05925-7 (PMC10313764; doi:10.1038/s41419-023-05925-7)
Supplement: Supplementary file 15 — Supplementary figure legends [file 41419_2023_5925_MOESM15_ESM.docx]

**Supplementary figure legends**

**Supplementary figure 1. BAPTA-AM blocks the intracellular calcium level**

**A**. BMDMs stained with Fluo-4 were incubated with or with BAPTA-AM (5 µM) for 30 min and analyzed by flow cytometry. Data are mean ± s.e.m. from n = 3 independent experiments. ***P < 0.001; two-way ANOVA. **B**. Fluo-4 stained BMDMs were treated with or without BAPTA-AM (5 µM) for 30 min, then incubated with PS beads, and observed by time-lapse microscopy. The video starts at 1 min before addition of PS beads and the arrowheads indicate PS beads being bound. Scale bar, 10 µm. **C**. The intensities of Fluo-4 were quantified using ImageJ. The initial intensities of Fluo-4 in control and BAPTA-AM treated phagocytes were set to 1 at time 0.

**Supplementary figure 2. Effects of intracellular calcium depletion on efferocytosis**

**A–C**. PMs (**A**), RAW264.7 cells (**B**), and J774A.1 cells (**C**) treated with BAPTA-AM were incubated with TAMRA-stained apoptotic cells for 15 min (**A**, **C**) or 1 h (**B**) and analyzed by flow cytometry. Data are mean ± s.e.m. from n = 3 independent experiments. *P < 0.05; **P < 0.01; two-tailed paired Student’s t test. **D**. J774A.1 cells treated with BAPTA-AM were incubated with Cy3-labeled PS beads for 15 min and analyzed by flow cytometry. Data are mean ± s.e.m. from n = 3 independent experiments. **P < 0.01; two-tailed paired Student’s t test. **E**. CellMask-stained BMDMs were treated with the indicated concentrations of BAPTA-AM, incubated with TAMARA-stained apoptotic cells at 4°C for 2 h, fixed, stained with DAPI, and observed by fluorescence microscopy (left). The number of bound apoptotic cells per phagocyte was quantified (right). Scale bar, 50 µm. Data are mean ± s.e.m. from n = 14, 15, and 12 randomly acquired images. NS, not significant; one-way ANOVA.

**Supplementary figure 3. Rac1 activation in phagocytes treated with BAPTA-AM**

**A**. J774A.1 cells expressing Raichu-Rac1 were incubated with or without BAPTA-AM (5µM), then incubated with PS beads, and observed by time-lapse confocal microscopy. The time starts at addition of PS beads. The asterisks indicate the PS beads being engulfed. Scale bar, 10 µm. **B**. The average intensities of FRET of phagocytes over time were quantified. Data are mean ± s.e.m. from n = 240 images acquired at each time points. NS, not significant; two-tailed unpaired Student’s t test. **C**. Schematic diagram illustrating the measurement of time required for internalization of the targets. The time starts when the phagocytic cup forms at the interface between PS beads and phagocytes, and ends when PS beads are completely surrounded by the plasma membrane and pulled into phagocytes.

**Supplementary figure 4. Effects of interfering with the CaM-MLCK-MLC signaling on efferocytosis**

**A**. BMDMs treated with W-7 or ML-7 were incubated with TAMRA-stained apoptotic thymocytes for 15 min and analyzed by flow cytometry. Data are mean ± s.e.m. from n = 3 independent experiments. *P < 0.05; one-way ANOVA. **B**. MLCK KO J774A.1 cells were incubated with TAMRA-stained apoptotic thymocytes for 15 min and analyzed by flow cytometry. Data are mean ± s.e.m. from n = 3 independent experiments. *P < 0.05; one-way ANOVA. **C**. MLCK KO J774A.1 cells were incubated with Cy5- and pHrodo-labeled PS beads for 15 min and analyzed by flow cytometry. Data are mean ± s.e.m. from n = 3 independent experiments. NS, not significant; **P < 0.01; two-way ANOVA. **D**. MLCK KO J774A.1 cells stained with CellMask were incubated with Cy3-labeled PS beads at 4°C for 2 h, fixed, stained with DAPI, and observed by microscopy (left). The number of bound PS beads per phagocyte was quantified (right). Scale bar, 50 µm. Data are mean ± s.e.m. from n = 20, 15, 12, and 13 randomly acquired images. NS, not significant; one-way ANOVA. **E**. MLCK KO J774A.1 cells stained with CellMask were incubated with PS beads for 10 min, fixed, stained with phalloidin, and observed by confocal microscopy. The intensities of F-actin across PS beads were measured using ImageJ. Distance 0 µm indicates the center of the PS bead. n = 36 beads for Ctrl and 47 beads for MLCK KO.

**Supplementary figure 5. Rac1 activation and F-actin formation in *Mertk^-/-^* BMDMs during efferocytosis.**

**A**. BMDMs derived from *WT* and *Mertk^-/-^* mice were incubated with apoptotic cells for 20 min, extensively washed, and lysed. The lysates were incubated with GST-tagged PAK-1 PBD fusion protein conjugated to glutathione agarose beads and bound proteins were detected by an anti-Rac1 antibody. The precipitated Rac1 was quantified as the ratio of precipitated Rac1 to the total Rac1. The numbers indicate the ratio of the precipitated Rac1 in *Mertk^-/-^* BMDMs to that in *WT* BMDMs. **B**. BMDMs derived from *WT* and *Mertk^-/-^* mice were incubated with apoptotic thymocytes for 20 min, fixed, stained with phalloidin, and observed by confocal microscopy. The intensities of F-actin across the targets were quantified using ImageJ. Distance 0 µm is the center of the apoptotic cell.
